# Spatially Dense 3D Facial Heritability and Modules of Co-heritability in a Father-Offspring Design

**DOI:** 10.3389/fgene.2018.00554

**Published:** 2018-11-19

**Authors:** Hanne Hoskens, Jiarui Li, Karlijne Indencleef, Dorothy Gors, Maarten H. D. Larmuseau, Stephen Richmond, Alexei I. Zhurov, Greet Hens, Hilde Peeters, Peter Claes

**Affiliations:** ^1^Department of Human Genetics, KU Leuven, Leuven, Belgium; ^2^Medical Imaging Research Center, University Hospitals Leuven, Leuven, Belgium; ^3^Department of Electrical Engineering, ESAT/PSI, KU Leuven, Leuven, Belgium; ^4^Research Group Experimental Otorhinolaryngology, Department of Neurosciences, KU Leuven, Leuven, Belgium; ^5^Forensic Biomedical Sciences, Department of Imaging and Pathology, KU Leuven, Leuven, Belgium; ^6^Applied Clinical Research and Public Health, School of Dentistry, College of Biomedical and Life Sciences, Cardiff University, Cardiff, United Kingdom; ^7^Murdoch Childrens Research Institute, Melbourne, VIC, Australia

**Keywords:** 3D imaging, (co-)heritability, face, ALSPAC, geometric morphometrics, spatially dense, modularity

## Abstract

**Introduction:** The human face is a complex trait displaying a strong genetic component as illustrated by various studies on facial heritability. Most of these start from sparse descriptions of facial shape using a limited set of landmarks. Subsequently, facial features are preselected as univariate measurements or principal components and the heritability is estimated for each of these features separately. However, none of these studies investigated multivariate facial features, nor the co-heritability between different facial features. Here we report a spatially dense multivariate analysis of facial heritability and co-heritability starting from data from fathers and their children available within ALSPAC. Additionally, we provide an elaborate overview of related craniofacial heritability studies.

**Methods:** In total, 3D facial images of 762 father-offspring pairs were retained after quality control. An anthropometric mask was applied to these images to establish spatially dense quasi-landmark configurations. Partial least squares regression was performed and the (co-)heritability for all quasi-landmarks (∼7160) was computed as twice the regression coefficient. Subsequently, these were used as input to a hierarchical facial segmentation, resulting in the definition of facial modules that are internally integrated through the biological mechanisms of inheritance. Finally, multivariate heritability estimates were obtained for each of the resulting modules.

**Results:** Nearly all modular estimates reached statistical significance under 1,000,000 permutations and after multiple testing correction (*p* ≤ 1.3889 × 10^-3^), displaying low to high heritability scores. Particular facial areas showing the greatest heritability were similar for both sons and daughters. However, higher estimates were obtained in the former. These areas included the global face, upper facial part (encompassing the nasion, zygomas and forehead) and nose, with values reaching 82% in boys and 72% in girls. The lower parts of the face only showed low to moderate levels of heritability.

**Conclusion:** In this work, we refrain from reducing facial variation to a series of individual measurements and analyze the heritability and co-heritability from spatially dense landmark configurations at multiple levels of organization. Finally, a multivariate estimation of heritability for global-to-local facial segments is reported. Knowledge of the genetic determination of facial shape is useful in the identification of genetic variants that underlie normal-range facial variation.

## Introduction

The human face is a complex trait displaying a strong genetic component (Kohn, 1991), as evidenced by remarkable facial similarity between identical twins, clear facial resemblances within families, geographic populations (Hopman et al., 2014) and the sexes (Claes et al., 2012b), and finally the distinctive facial features associated with particular genetic conditions (Hammond, 2007; Baynam et al., 2013). This suggests that inter-individual variation in craniofacial morphology is largely determined by genetic variation, most likely in combination with diverse environmental influences.

Studies on craniofacial heritability provide insight into the relative contribution of genetic versus environmental effects on craniofacial parameters. These studies highlight similarities as well as differences in patterns of inheritance, resulting from differences in the study population (sample size, age of assessment, sex, ethnicity), in the capturing technique (2D or 3D), in the way facial shape is measured and finally in the statistical methods yielded. Most craniofacial heritability studies are performed on twin and family (siblings or parent-offspring) databases and many of these apply 2D imaging techniques, limiting their results due to the loss of information when quantifying the facial phenotype in two dimensions only (Tables 1, 2). More recently, 3D imaging techniques are being used to characterize facial morphology (Tables 1–3). All studies, 2D as well as 3D, start from sparse descriptions of facial shape using a limited set of landmarks, with one recent exception that uses spatially dense landmarking instead (Tsagkrasoulis et al., 2017). Variation in these landmarks is simplified by the projection of multivariate landmark configurations onto principal components (PC) or by measuring geometric features such as distances, curvature, ratios and/or angles from the landmarks. Subsequently, a heritability score is computed for each individual PC or geometric feature separately. However, any preselection of individual PCs or geometric features fails to capture the full-range of facial variations as combinations of these measurements are not considered. Finally, heritability studies today do not investigate co-inheritance between different facial features.

**Table 1:** Literature review of craniofacial heritability – twin studies.

Study sample	Measures and Techniques	Effect	Reference
**NUMBER** 79 twin pairs (33 MZ, 46 DZ) **SEX DISTRIBUTION** MZ: 17 male, 16 female DZ: 14 male, 14 female, 18 male-female **AGE DISTRIBUTION** 9-16 years mean 12.1 years **ETHNIC BACKGROUND** Belgium	**CAPTURING TECHNIQUE** 2D lateral cephalograms **STATISTICAL ANALYSIS** Model-fitting **FACIAL PHENOTYPE** 23 measures (linear, angular)	**RANGE** 45.3–91.2% **GENETIC DETERMINATION** Total anterior facial height (male) Anterior cranial base Nasion horizontal Incisor superior vertical Incisor inferior vertical **ENVIRONMENTAL INFLUENCE** Saddle angle Gonial angle Relative sagittal position of the mandible and maxilla to the anterior cranial base **NOTES** *h*^2^: vertical > horizontal measures	Carels et al., 2001

**NUMBER** 26 twin pairs (10 MZ, 16 DZ) **SEX DISTRIBUTION** MZ: 5 male, 5 female DZ: 3 male, 7 female, 6 male-female **AGE DISTRIBUTION** 6-42 years Mean 12 years **ETHNIC BACKGROUND** Mixed ethnicity	**CAPTURING TECHNIQUE** 3D facial scans **STATISTICAL ANALYSIS** Intrapair differences (no *h*^2^-values) **FACIAL PHENOTYPE** 18 landmarks 28 measures (linear) Surface measures (curvature)	**GENETIC DETERMINATION** Nasal height and width Left eye width Intercanthal width **NOTES** *h*^2^: vertical > horizontal measures High *h*^2^ for central midfacial structures (triangular area encompassing the nose, orbital rims, intercanthal area)	Naini and Moss, 2004.

**NUMBER** 20 twin pairs (10 MZ, 10 DZ) **SEX DISTRIBUTION** MZ: 5 male, 5 female DZ: 3 male, 7 female **AGE DISTRIBUTION** mean 12 years	**CAPTURING TECHNIQUE** 3D facial scans **STATISTICAL ANALYSIS** Surface shape analysis (no h^2^-values) **FACIAL PHENOTYPE** Surface measures (curvature)	**GENETIC DETERMINATION** Brow ridges Nasion Infraorbital margins **ENVIRONMENTAL INFLUENCE** Chin Cheeks Lips	Moss, 2006

**NUMBER** 50 twin pairs (25 MZ, 25 DZ) **SEX DISTRIBUTION** MZ: 13 male, 12 female DZ: 13 male, 12 female **AGE DISTRIBUTION** 13.4–20.1 years mean 16.4 years **ETHNIC BACKGROUND** Iran	**CAPTURING TECHNIQUE** 2D lateral cephalograms **STATISTICAL ANALYSIS** classical correlation analysis **FACIAL PHENOTYPE** 33 measures (linear, angular)	**RANGE** 6–162% **GENETIC DETERMINATION** Gonial angle Saddle angle Total anterior facial height Lower anterior facial height Relative sagittal position of the mandible Relative sagittal position of the maxilla Anterior cranial base **ENVIRONMENTAL INFLUENCE** Upper anterior facial height Mandibular body length **NOTES** *h*^2^: vertical > horizontal measures High heritability found in lower third of the face Low heritability for dento-alveolar variables	Amini and Borzabadi-Farahani, 2009

**NUMBER** 21 twin pairs (10 MZ, 11 DZ) **SEX DISTRIBUTION** Same-sex twins **AGE DISTRIBUTION** 5–12 years Mean 9.3 years **ETHNIC BACKGROUND** United States Caucasian	**CAPTURING TECHNIQUE** 3D facial scans **STATISTICAL ANALYSIS** Classical correlation analysis **FACIAL PHENOTYPE** 13 landmarks 17 measures (PC)	**RANGE** 90–100% **GENETIC DETERMINATION** Breadth of orbital and nasal structures Nasal length, breadth and projection Upper lip height and projection **NOTES** High *h*^2^ for central midfacial structures	Weinberg et al., 2013

**NUMBER** 37 twin pairs (19 MZ, 18 DZ) **SEX DISTRIBUTION** MZ: 9 male, 10 female DZ: 7 male, 3 female, 8 male-female **AGE DISTRIBUTION** 15.5 years **ETHNIC BACKGROUND** United Kingdom (ALSPAC)	**CAPTURING TECHNIQUE** 3D facial scans **STATISTICAL ANALYSIS** Shape analysis (no *h*^2^-values) **FACIAL PHENOTYPE** 21 landmarks Surface measures	**GENETIC DETERMINATION** Supraorbital and infraorbital ridges Forehead, lower lip and nasal bridge (males) Eyes, philtrum and lower part of the cheeks (females) **ENVIRONMENTAL INFLUENCE** Lower third of the face **NOTES** High *h*^2^ for central midfacial structures	Djordjevic et al., 2013

**NUMBER** 141 twin pairs (90 MZ, 51 DZ) **SEX DISTRIBUTION** MZ: 29 male, 61 female DZ: 20 male, 31 female **AGE DISTRIBUTION** 15.3–39.6 years Mean 21.7 years **ETHNIC BACKGROUND** Lithuania	**CAPTURING TECHNIQUE** 2D lateral cephalograms **STATISTICAL ANALYSIS** Model-fitting **FACIAL PHENOTYPE** 39 measures (linear, angular)	**RANGE** 20–84% **GENETIC DETERMINATION** Incision inferior to nasion–basion distance Sagittal position of the mandible Gonial angle **ENVIRONMENTAL INFLUENCE** Mandibular body length Ramus width and height **NOTES** *h*^2^: horizontal > vertical measures h^2^: form (angular) > size (linear) h^2^: skeletal > dentoalveolar measures High *h*^2^ for the ‘polygon of the facial profile similarity’ (area determined by the angles SNB, NSAr, ArGoMe)	Šidlauskas et al., 2016

**ETHNIC BACKGROUND** United Kingdom (TwinsUK)	**CAPTURING TECHNIQUE** 3D facial images **STATISTICAL ANALYSIS** model-fitting **FACIAL PHENOTYPE** 21 landmarks 210 pairwise distances	**RANGE** 0–66% **GENETIC DETERMINATION** nasal region mouth	de Jong et al., 2016.

**NUMBER** 604 twin pairs (263 MZ, 341 DZ) 172 unpaired twins (75 MZ, 97 DZ) **SEX DISTRIBUTION** Female twins	**CAPTURING TECHNIQUE** 3D facial images **STATISTICAL ANALYSIS** Classical correlation analysis	**RANGE** Facial form (uPC): 38.8–78.5% Facial shape (sPC): 30.5–84.8%	Djordjevic et al., 2016
**AGE DISTRIBUTION** 23.6-86.5 years Mean 58.8 years **ETHNIC BACKGROUND** United Kingdom (TwinsUK)	**FACIAL PHENOTYPE** 51 landmarks 1317 measures (linear, scaled PCs, unscaled PCs)	**GENETIC DETERMINATION** Lips prominence Inter-ocular distance Facial size (height) Nasal width, prominence & height **ENVIRONMENTAL INFLUENCE** Mandibular ramus height Horizontal facial asymmetry	

**NUMBER** 476 twin pairs (197 MZ, 279 DZ) **SEX DISTRIBUTION** Female twins **AGE DISTRIBUTION** Mean 59.3 years **ETHNIC BACKGROUND** United Kingdom (TwinsUK)	**CAPTURING TECHNIQUE** 3D facial images **STATISTICAL ANALYSIS** Model-fitting **FACIAL PHENOTYPE** 4,096 landmarks 20 distances (Euclidean, Geodesic) 16,384 surface measures (curvature)	**RANGE** 0–78.9% **GENETIC DETERMINATION** Chin Nasal region Nasolabial folds Nasion Upper lips Zygomatic bones Inner canthi **ENVIRONMENTAL INFLUENCE** Orbits Lips	Tsagkrasoulis et al., 2017

**NUMBER** 1,567 individuals **SEX DISTRIBUTION** female twins **AGE DISTRIBUTION** mean 59.3 years **ETHNIC BACKGROUND** United Kingdom (TwinsUK)	**CAPTURING TECHNIQUE** 3D facial images **STATISTICAL ANALYSIS** classical correlation analysis **FACIAL PHENOTYPE** PCs for orbital and profile subregion	**RANGE** 76.1–81.5% **GENETIC DETERMINATION** eyes subregion profile subregion	Crouch et al., 2018

**NUMBER** 200 twin pairs (37 MZ, 163 DZ) **ETHNIC BACKGROUND** United Kingdom (TwinsUK)	**CAPTURING TECHNIQUE** 3D facial images **STATISTICAL ANALYSIS** Model-fitting **FACIAL PHENOTYPE** 225 measures (coordinates, distances, areas, angles)	**RANGE** 0–87% **GENETIC DETERMINATION** Area: (left) corner mouth, (left) alae nasi and (left) outer corner eye Distance: (right) inner corner eye, (left) alae nasi Area: (left) corner mouth, (left) alae nasi & (left) outer corner eye *x*-coordinate: (left) alae nasi	de Jong et al., 2018

**NUMBER** 26 twin pairs (13 MZ, 13 DZ) **SEX DISTRIBUTION** MZ: 7 male, 6 female DZ: 7 male, 6 female **AGE DISTRIBUTION** Mean 39 years **ETHNIC BACKGROUND** Korea	**CAPTURING TECHNIQUE** 2D lateral cephalograms **STATISTICAL ANALYSIS** Classical correlation analysis **FACIAL PHENOTYPE** 23 landmarks 47 measures (linear, angular, ratio)	**RANGE** -131.8–219% **GENETIC DETERMINATION** Horizontal relationship between maxilla, mandible and anterior cranial base ° Vertical ratios of anterior facial height shape of cranial base Location of occlusal plane within skeletal framework Vertical relationship among cranial base, palatal plane and mandibular plane ° Lower gonial angle Mandibular body length **ENVIRONMENTAL INFLUENCE** Anterior and posterior facial height Ramus height **NOTES** *h*^2^: horizontal > vertical > mandible > cranial base > dental measures	Kim et al., 2018.

*The first column (‘Study sample’) contains information on the study population. The second column (‘Measures and Techniques’) specifies the methodology. The third column (‘Effect’) summarizes the most important findings of the study (see also column 4 ‘Reference’). Heritability was considered to be low if *h*^2^< 35% (i.e., ‘environmental influence’) and moderate to high if *h*^2^ > 35% and *h*^2^ > 65%, respectively (i.e., ‘genetic determination’). MZ, monozygotic; DZ, dizygotic.*

**Table 2:** Literature review of craniofacial heritability – family studies.

Study sample	Measures and techniques	Effect	Reference
**Siblings**

**NUMBER** 138 siblings **SEX DISTRIBUTION** 68 males, 70 females **AGE DISTRIBUTION** Mean 23 years **ETHNIC BACKGROUND** Turkey	**CAPTURING TECHNIQUE** 2D lateral cephalograms **STATISTICAL ANALYSIS** Model-fitting **FACIAL PHENOTYPE** 12 measures (soft-tissue, ratio)	**RANGE** 30–109% **GENETIC DETERMINATION** Total depth index Soft-tissue chin thickness Merrifield angle Holdaway angle Soft-tissue facial angle Upper to lower facial height **NOTES** *h*^2^: depth > vertical measures	Baydaş et al., 2007

**Parent-offspring**

**NUMBER** 363 6-year-olds 182 16-year-olds **SEX DISTRIBUTION** 6y: 184 males, 179 females 16y: 97 males, 85 females **ETHNIC BACKGROUND** Iceland	**CAPTURING TECHNIQUE** 2D lateral cephalograms **STATISTICAL ANALYSIS** Regression analysis **FACIAL PHENOTYPE** 22 landmarks 33 measures (linear, angular, ratio)	**RANGE** F–S: (6y) -28–62%; (16y) -47–98% M–S: (6y) -33–65%; (16y) -26–107% F–D: (6y) -54–77%; (16y) -33–87% M–D: (6y) -47–83%; (16y) -63–104% **GENETIC DETERMINATION** Position of the lower jaw Anterior and posterior facial height Cranial base dimensions Nasal bone length and prominence **ENVIRONMENTAL INFLUENCE** Dental variables **NOTES** *h*^2^: daughters > sons *h*^2^: 16-year-olds > 6-year-olds	Johannsdottir et al., 2005

**NUMBER** 24 families **AGE DISTRIBUTION** children: 17-35 years parents: 35-65 years **ETHNIC BACKGROUND** Saudi Arabia	**CAPTURING TECHNIQUE** 2D lateral cephalograms **STATISTICAL ANALYSIS** regression analysis **FACIAL PHENOTYPE** 15 landmarks 28 measures (linear, angular, ratio)	**RANGE** F–S: 1–147% M–S: 2–85% F–D: 11–118% M–D: 1–113% **GENETIC DETERMINATION** mandibular variables (°) facial height dimensions mandibular body length **NOTES** h^2^: daughters > sons h^2^: father-offspring > mother-offspring h^2^: linear > angular measures h^2^: mandibular > maxillary variables	AlKhudhairi and AlKofide, 2010

**NUMBER** 140 individuals from 35 families **AGE DISTRIBUTION** geq 16 years **ETHNIC BACKGROUND** India	**CAPTURING TECHNIQUE** 2D digital photographs **STATISTICAL ANALYSIS** Correlation analysis (no *h*^2^-values) **FACIAL PHENOTYPE** 27 measures (linear, ratio)	**GENETIC DETERMINATION** Mandibular position Chin prominence Nasal prominence & width Lip length at philtrum Total facial height Lip prominence **ENVIRONMENTAL INFLUENCE** Nose and lip form **NOTES** *h*^2^: daughters > sons	Lahoti et al., 2013

**NUMBER** 762 father-offspring pairs **SEX DISTRIBUTION** 358 males, 404 females **AGE DISTRIBUTION** Children: 15.5 years Fathers: 40–75 years	**CAPTURING TECHNIQUE** 3D facial scans **STATISTICAL ANALYSIS** Multivariate regression analysis **FACIAL PHENOTYPE** 7,160 landmarks 63 facial segments	**RANGE** Sons: 34–82% Daughters: 32–72% **GENETIC DETERMINATION** Global face Upper facial part Nose	Current study

**ETHNIC BACKGROUND** United Kingdom (ALSPAC)		Orbital region **ENVIRONMENTAL INFLUENCE** Cheeks	
		Small segments around philtrum **NOTES** *h*^2^: sons > daughters	

**Nuclear and extended families**

**NUMBER** 1,918 individuals from 342 families **SEX DISTRIBUTION** Children: 598 males, 464 females Parents: 390 males, 466 females **AGE DISTRIBUTION** 6–72 years Mean 21.5 years **ETHNIC BACKGROUND** India	**CAPTURING TECHNIQUE** Direct anthropometric measurements **STATISTICAL ANALYSIS** Model-fitting **FACIAL PHENOTYPE** 23 measures (linear, craniofacial, soft-tissue)	**RANGE** 25–61% **GENETIC DETERMINATION** Bizygomatic breadth Nasal breadth and height Head breadth and length Facial height **NOTES** *h*^2^: craniofacial > linear measures *h*^2^: breadth measures = circumference	Arya et al., 2002

**NUMBER** 1,406 individuals from 357 families **SEX DISTRIBUTION** 733 males, 673 females **AGE DISTRIBUTION** 17–90 years **ETHNIC BACKGROUND** Russia	**CAPTURING TECHNIQUE** Direct anthropometric measurements **STATISTICAL ANALYSIS** Classical correlation analysis model-fitting **FACIAL PHENOTYPE** 10 measures, 2 latent factors (f)	**RANGE** 52–72% **GENETIC DETERMINATION** Horizontal component (f) Bizygomatic breadth Minimum frontal breadth Head breadth and length Vertical component (f) Nasal height nasion **NOTES** *h*^2^: horizontal > vertical measures	Ermakov et al., 2005

**NUMBER** 298 subjects from 54 families **SEX DISTRIBUTION** 127 males, 171 females	**CAPTURING TECHNIQUE** 3D models of the skull **STATISTICAL ANALYSIS** Model-fitting	**RANGE** 0–86.7% **GENETIC DETERMINATION** External alveolar breadth	Carson, 2006
**AGE DISTRIBUTION** Adults **ETHNIC BACKGROUND** Austria, Hallstatt population	**CAPTURING TECHNIQUE** 3D models of the skull **STATISTICAL ANALYSIS** Model-fitting **FACIAL PHENOTYPE** 58 landmarks 33 measures (linear)	Nasal height Bimaxillary breadth Nasion-prosthion height **ENVIRONMENTAL INFLUENCE** Bifrontal breadth Nasal breadth Biorbital breadth **NOTES** *h*^2^: vertical > horizontal measures *h*^2^: neurocranial > facial measures	

**NUMBER** 1,263 individuals from 373 families **SEX DISTRIBUTION** 686 males, 577 females **AGE DISTRIBUTION** 18–81 years **ETHNIC BACKGROUND** India	**CAPTURING TECHNIQUE** Direct anthropometric measurements **STATISTICAL ANALYSIS** Classical correlation analysis Model-fitting **FACIAL PHENOTYPE** 11 measures, 2 latent factors (f)	**RANGE** 41–83% **GENETIC DETERMINATION** Nasal height nasion Vertical head factor (f) Horizontal head factor (f) Bizygomatic breadth Minimum frontal breadth Physiognomic super facial height **NOTES** *h*^2^: horizontal = vertical measures	Karmakar et al., 2007

**NUMBER** 474 individuals from 119 families **SEX DISTRIBUTION** 238 males, 236 females	**CAPTURING TECHNIQUE** Direct anthropometric measurements **STATISTICAL ANALYSIS** Classical correlation analysis Model-fitting	**RANGE** 52–80% **GENETIC DETERMINATION** Head breadth Bizygomatic breadth	Jelenkovic et al., 2008

**AGE DISTRIBUTION** 17–72 years **ETHNIC BACKGROUND** Belgium	**FACIAL PHENOTYPE** 14 measures, 4 latent factors (f)	Horizontal head factor 1 (f) Horizontal facial factor (f) External biocular breadth Horizontal head factor 2 (f) Nose breadth	
		**NOTES** *h*^2^: horizontal > vertical measures *h*^2^: facial > head phenotypic measures	

**NUMBER** 607 individuals from 90 families **SEX DISTRIBUTION** 328 males, 279 females **AGE DISTRIBUTION** 13–75.5 years (observation closest to the participant’s 18th birthday was chosen for analysis) **ETHNIC BACKGROUND** Ohio (Fels Longitudinal Study) European ancestry	**CAPTURING TECHNIQUE** 2D lateral cephalograms **STATISTICAL ANALYSIS** Model-fitting **FACIAL PHENOTYPE** 10 landmarks 10 measures (linear, angular)	**RANGE** 34–71% **GENETIC DETERMINATION** Anterior basicranial length (S-N) Sella-vertex (ectocranial) Basocranial flexion (Ba-S-N°) Sella-sphenoethmoidale Facial positioning (S-N-A°) Total basicranial length (Ba-N) **ENVIRONMENTAL INFLUENCE** Posterior base (Ba-S)	Sherwood et al., 2008

**NUMBER** 355 subjects **SEX DISTRIBUTION** 211 males, 144 females **AGE DISTRIBUTION** Adults **ETHNIC BACKGROUND** Austria, Hallstatt population	**CAPTURING TECHNIQUE** 3D models of the skull **STATISTICAL ANALYSIS** Model-fitting **FACIAL PHENOTYPE** 65 landmarks 58 measures (linear)	**RANGE** 0–43% **GENETIC DETERMINATION** Nasal height and length Orbital breadth (frontomalare orbitale) Zygomatic height Orbital length **ENVIRONMENTAL INFLUENCE** Nasal breadth Zygomatic breadth **NOTES** *h*^2^: basicranial = neurocranial = facial	Martínez-Abadías et al., 2009

**NUMBER** 509 individuals from 122 families **SEX DISTRIBUTION** 251 males, 258 females **AGE DISTRIBUTION** 13–72 years **ETHNIC BACKGROUND** Belgium	**CAPTURING TECHNIQUE** Direct anthropometric measurements **STATISTICAL ANALYSIS** Model-fitting **FACIAL PHENOTYPE** 18 craniofacial measures (skeletal, soft-tissue)	**RANGE** 46–72% **GENETIC DETERMINATION** External biocular breadth Lips height Head breadth Minimum frontal breadth Bigonial breadth Physiognomic facial height Bizygomatic breadth	Jelenkovic et al., 2010
		**NOTES** *h*^2^: skeletal > soft-tissue measures	

**NUMBER** 229 individuals from 38 families **SEX DISTRIBUTION** 94 males, 135 females **AGE DISTRIBUTION** Children: mean 36.0 years Parents: mean 55.2 years **ETHNIC BACKGROUND** Korea	**CAPTURING TECHNIQUE** 2D digital photographs **STATISTICAL ANALYSIS** Model-fitting **FACIAL PHENOTYPE** 25 landmarks 14 measures, 3 latent factors (f)	**RANGE** 25–61% **GENETIC DETERMINATION** Intercanthal width Lower face portion (f) Nose width Orbital region (f) Vertical length (f) **ENVIRONMENTAL INFLUENCE** Mouth width Lower facial height	Kim et al., 2013

**NUMBER** 1,379 individuals from 127 families **SEX DISTRIBUTION** 655 males, 724 females	**CAPTURING TECHNIQUE** 2D lateral cephalograms **STATISTICAL ANALYSIS** Model-fitting	**RANGE** 10–60% **GENETIC DETERMINATION** Nasion-sella-basion (°) Pogonion to nasion-basion	Šešelj et al., 2015

**AGE DISTRIBUTION** 8–95 years (observation closest to the participant’s 18th birthday was chosen for analysis) **ETHNIC BACKGROUND** Ohio (Fels Longitudinal Study) European ancestry	**FACIAL PHENOTYPE** 48 landmarks 75 measures (linear, angular)	Gonial angle Lower facial height Sella to nasion **ENVIRONMENTAL INFLUENCE** Molar relation Palatal plane Ramus position Lip protrusion **NOTES** *h*^2^: neurocranial > basicranial and facial measures	

*The first column (‘Study sample’) contains information on the study population. The second column (‘Measures and Techniques’) specifies the methodology. The third column (‘Effect’) summarizes the most important findings of the study (see also column 4 ‘Reference’). Heritability was considered to be low if *h*^2^< 35% (i.e., ‘environmental influence’) and moderate to high if *h*^2^ > 35% and *h*^2^ > 65%, respectively (i.e., ‘genetic determination’). F, father; M, mother; S, son; D, daughter.*

**Table 3:** Literature review of craniofacial heritability – population studies.

Study sample	Measures and techniques	Effect	Reference
**NUMBER** 3480 individuals **SEX DISTRIBUTION** 44.4% males 55.6% females **AGE DISTRIBUTION** 3–21 years 70% in 7–12 age bracket **ETHNIC BACKGROUND** Tanzania Bantu children, Mwanza region	**CAPTURING TECHNIQUE** 3D facial scans **STATISTICAL ANALYSIS** Model-fitting (GCTA) >15 million common SNPs **FACIAL PHENOTYPE** 29 landmarks 38 measures (PC, linear, size)	**RANGE** 28.3–66.9% **GENETIC DETERMINATION** Nasal root shape, mouth width Total facial width Allometry Centroid size Nasion-midendocanthion distance Nasal width Nose width, mandible height Total facial shape Midfacial landmark network around nose and mouth **ENVIRONMENTAL INFLUENCE** Upper vermilion height Nasal width, maxillary prognathism Lower lip height Chin height, nasion protrusion **NOTES** *h*^2^: horizontal > vertical and depth measures >90% of the narrow-sense h^2^ can be explained by common genetic variation High absolute genetic correlations between most traits: large overlap in underlying genetic loci	Cole et al., 2017

*The first column (‘Study sample’) contains information on the study population. The second column (‘Measures and Techniques’) specifies the methodology. The third column (‘Effect’) summarizes the most important findings of the study (see also column 4 ‘Reference’). Heritability was considered to be low if *h*^2^< 35% (i.e., ‘environmental influence’) and moderate to high if *h*^2^ > 35% and *h*^2^ > 65%, respectively (i.e., ‘genetic determination’).*

In this work, we present a global-to-local analysis of heritability and co-heritability of multivariate facial modules in a father-offspring design. First, we establish spatially dense quasi-landmarks representing complete facial shape and compute 3D landmark heritability as well as 3D pairwise landmark co-heritability. Subsequently, we apply hierarchical spectral clustering to these values and define 63 modules (multiple 3D landmarks grouped together) of co-inheritance, which when analyzed as groups, provide a multivariate estimation of heritability for various facial segments, ranging from the full face (global) to smaller facial regions (local). Finally, in the discussion we embed our results in an elaborate overview of related craniofacial heritability studies published from the year 2000 and onward (Tables 1–3).

## Materials and Methods

### Sample and Recruitment

Data were collected from the Avon Longitudinal Study of Parents and Children (ALSPAC), a UK-based birth cohort study designed to explore genetic and environmental influences on child health and development. In brief, all pregnant women with an expected delivery date between 1 April 1991 and 31 December 1992 inclusive, were eligible to participate in ALSPAC. A total of 14,541 pregnant women were recruited as part of Phase I (Boyd et al., 2013; Fraser et al., 2013). Detailed information and biological samples have been collected from these women and their offspring at various time points. The study website contains details of all the data that is available through a fully searchable data dictionary^1^.

3D facial surface scans were obtained for 4,731 adolescents at the 15-year-old follow-up clinic. A total of 3,663 fathers, from the ALSPAC cohort, were also invited to take part in the study. Additional phenotypic measures included demographic descriptors (e.g., sex, age, self-reported ethnicity), basic physical characteristics (e.g., height, weight) and information regarding the pregnancy. Children and their corresponding fathers could be linked by a unique pregnancy identifier, which resulted in 992 pairs (979 first and 13 second born). Participants with missing information on sex, age, height, weight (*N* = 21) and self-reported ethnic background (*N* = 45) were excluded from this study.

The current study (B2409: “Exploring the heritability of facial features in fathers and offspring using spatially dense geometric morphometrics”) was approved by the ALSPAC Ethics and Law Committee and the Local Research Ethics Committees. Written informed consent was obtained from all children and their fathers in accordance with the Declaration of Helsinki.

### 3D Facial Imaging and Preprocessing

3D facial images were captured with two high-resolution Konica Minolta Vivid (VI900) laser scanners (Konica Minolta Sensing Europe Company, Milton Keynes, United Kingdom). The set of left and right facial images of each scanned subject were processed, registered and merged to generate 3D images of the face (Toma et al., 2008). Prior to the scanning procedure, participants were asked to remain still and present a neutral facial expression.

3D images were imported into an in-house scan cleaning program and hair, ears and any dissociated polygons were removed. Rough facial orientation was established by indicating five crude positioning landmarks, followed by non-rigidly mapping^2^ an anthropometric mask (AM) onto the images (Snyders et al., 2014; Claes et al., 2018). The AM is a predefined surface template covering the facial area of interest and its mapping results in the standardization of image data from all individuals by creating homologous spatially dense (∼7,160) quasi-landmark configurations (Claes et al., 2012a). Subsequently, generalized Procrustes analysis (GPA) was performed to eliminate differences in position, orientation and size of both original and reflected configurations combined, where the latter could be constructed by changing the sign of the x-coordinate (Claes et al., 2011). The average of an original and its reflected configuration constitutes the symmetric component, while the difference between the two constitutes the asymmetric component. Because faces display bilateral symmetry, aspects of symmetry and asymmetry are preferably considered separately when examining facial shape (Claes et al., 2012b). Although patterns of asymmetry may be informative, in this work we concentrate on the symmetric component only.

### Facial Quality Control

Outlier faces were detected by establishing *z*-scores for each face as described by Claes et al. (2018). Manual inspection of faces with a *z*-score equal to or larger than 2 led to the removal of imaging and mapping errors (*N* = 24) and participants displaying non-neutral facial expressions (*N* = 19) or whose images were obstructed by facial hair (*N* = 85). A further reduction was done by excluding participants with self-reported non-European ancestry (*N* = 28) and by randomly selecting one sibling from each multiple pregnancy (*N* = 8), so that only one child per family was included. A total of 762 father-child pairs were retained for analysis, including 358 sons and 404 daughters. Distribution statistics for age and BMI can be found in Supplementary Table S1 for all three cohorts (sons, daughters, and fathers). Lastly, GPA was applied to superimpose and symmetrize the facial shapes.

### Regression-Based Heritability Estimation

Patterns of heritability can be explored from the regression of offspring on parents (Falconer and Mackay, 1996). A multivariate generalization was proposed by Monteiro et al. (2002) based on the Procrustes distance. The Procrustes distance serves as a measure of shape difference and was used to compute a multivariate shape coefficient of determination (*R*^2^), which could then be transformed to a regression coefficient, reflecting the shape heritability. An extension for the use of high-dimensional data is provided here. Partial least squares regression (PLSR; function plsregress from Matlab 2017b) was performed to predict facial variation in children given the father’s facial variation. PLSR was preferred for this task because it allows to work with two blocks of multivariate and high-dimensional data. Furthermore, PLSR, in contrast to an ordinary multiple regression, is not constrained by collinearity in the data, which for 3D landmarks is practically always present (Zelditch et al., 2004; Tøndel et al., 2011). In essence, PLSR decomposes the dependent and independent variables into pairs of (unobserved) latent variables by maximizing the covariance between the two, which makes it a better prediction model (Zelditch et al., 2004; Shrimpton et al., 2014). Transformation of the variance explained by the regression model (*R*^2^) to a multivariate regression coefficient was done according to Monteiro et al. (2002). Given a one-parent one-offspring design, the heritability can directly be estimated by multiplying the regression coefficient by two (Falconer and Mackay, 1996). Note that the reported *R*^2^-coefficient was equal to the multivariate shape coefficient of determination defined in terms of the Procrustes distance.

### Facial Segmentation and Modules of Co-inheritance

First, the symmetrized facial shapes were adjusted for the confounding effects of age, sex, and BMI using PLSR (Claes et al., 2018). This was done for fathers and children separately. Next, each quasi-landmark was used as a 3D shape variable [x,y,z] and multivariate heritability estimates were obtained as described in Section “Regression-Based Heritability Estimation.” For both sons and daughters combined, a quasi-landmark of the offspring was regressed on the corresponding as well as all other quasi-landmarks of the father. This was done for all the quasi-landmarks in the offspring and the result was a squared similarity matrix (N × N, with N the number of quasi-landmarks). The heritability of each quasi-landmark was located on the diagonal of this matrix, while the co-inheritance between different quasi-landmarks was located in the off-diagonal elements. The mean squared error (MSE), generated by the function plsregress, was used to evaluate the quality of the PLSR model.

The symmetrized similarity matrix was used as input to perform a hierarchical spectral clustering with five levels. A detailed description of the clustering technique is provided by Claes et al. (2018). Quasi-landmarks with strong co-inheritance were grouped together into a series of facial segments or modules, rather than the clustering of highly correlated quasi-landmarks as observed in the structural segmentation (Claes et al., 2018). In order to assess whether differences existed between both segmentations, we computed the normalized mutual information (NMI). NMI values range from 0 to 1, with high values indicating a substantial overlap between two alternative segmentations (Claes et al., 2018).

All quasi-landmarks of the resulting 63 modules were subjected to a new GPA for both children and fathers combined, thereby creating a shape space for each of the facial modules. These shape spaces were constructed independently of the other modules and their relative positioning within the full face, so that only shape information was retained. Yet, integration of the modules was preserved through the hierarchical construction. Subsequently, each shape space was spanned by an orthogonal basis of PCs and parallel analysis was applied to determine the number of significant PCs contributing to facial shape. In contrast to related work using PCs, the resulting PCs are always used together and never individually, to provide a single multivariate description of modular shape variations.

### Facial Heritability Per Module

Sons and daughters were treated separately to estimate the heritability and co-heritability for all facial modules (cf. Regression-Based Heritability Estimation). The multidimensional nature of shape was preserved by performing PLSR on all PCs simultaneously and the quality of the regression model was assessed through the MSE. Labels between fathers and children were randomized and 1,000,000-fold permutation tests were undertaken for all 63 modules to determine any significant differences. The significance threshold correcting for the multiple-testing burden was determined at α = 1.3889 × 10^-3^ (i.e., 0.05/36), corresponding to an adjustment for the number of effective independent tests. The effective number was computed from the eigenvalues of the correlation matrix containing pairwise multivariate correlations of all 63 modules (Li and Ji, 2005). The reduction in effective tests was expected because of the dependency between neighboring quasi-landmarks and the hierarchical and overlapping construction of the facial modules (Claes et al., 2018). Finally, we experimentally determined the extent to which the heritability estimation is affected by the sample size in our multivariate approach by computing the heritability of randomly generated subsamples of different sizes.

The construction of the modular shape spaces on data of children and fathers altogether allowed us to evaluate whether similar patterns of variation in fathers and children were correlated, e.g., does variation in the nasal breadth of children reflect the same pattern of variation in fathers. When corresponding modules in children–father pairs were considered, shape variations encoded by the extracted latent variables each represented a particular direction within the same shape space. Both directions could be depicted graphically by creating morphs and by plotting the normal displacement map between the upper and lower extremes.

## Results

### 3D Landmark (Co-)heritability and Modules of Co-inheritance

**FIGURE 1:**
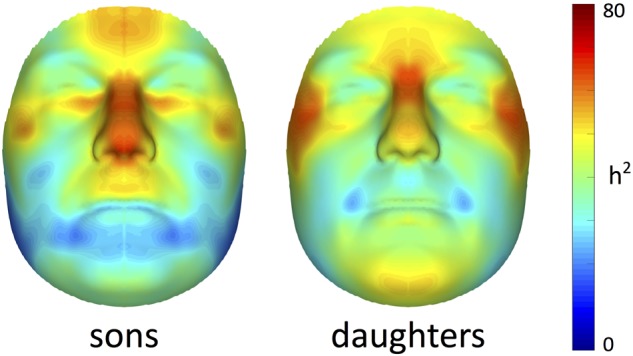
Facial heritability maps. 3D landmark heritability (%) for sons and daughters as obtained from the regression on fathers. The red-blue spectrum represents regions of high and low heritability, respectively. The maximum value was set to 80% for visualization purposes.

Facial heritability maps of both sons and daughters are shown in Figure 1. As expected, these maps were symmetric and coherent, without abrupt changes in the estimates of neighboring quasi-landmarks. Regions in the face with the greatest heritability included the areas encompassing the nasion and zygomas, as well as the nose and forehead. Differences between both cohorts were found in the lower part of the face. For example, higher estimates were obtained in the chin area for daughters compared with sons, while the latter showed higher heritability estimates in the philtrum area. All MSE values were close to zero (Supplementary Figure S1), yet slightly more variation was observed around the chin, nose and forehead.

**FIGURE 2:**
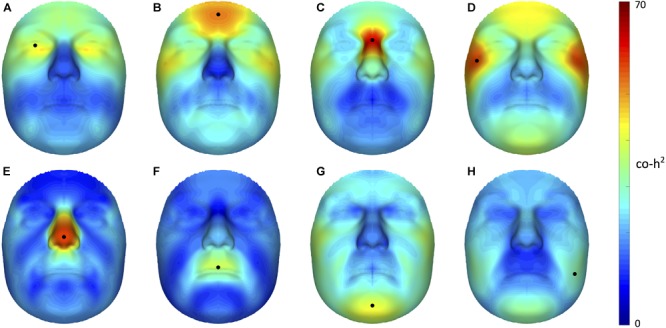
Facial maps of co-heritability. 3D pairwise landmark co-heritability (%) of the **(A)** orbital region, **(B)** forehead, **(C)** nasion, **(D)** zygomas, **(E)** nasal tip **(F)** upper lip, **(G)** chin and **(H)** cheeks. Landmarks of interest are indicated by a black dot, each representing the quasi-landmark in fathers that was used to predict facial variation in children for the corresponding as well as all other quasi-landmarks. The red-blue spectrum represents regions of high and low co-heritability, respectively. The maximum value was set to 70% for visualization purposes.

**FIGURE 3:**
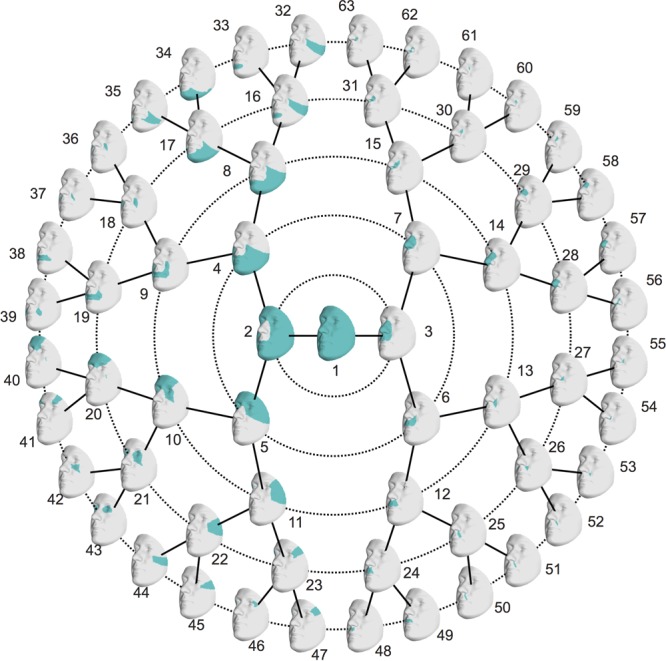
Modules of co-inheritance. Hierarchical facial segmentation of the study cohort, resulting from the grouping of quasi-landmarks with strong co-inheritance (*N* = 762 father-offspring pairs). Segments are colored in blue. Facial shape variation is covered at five different levels of detail, with global shape variations located in the center (L_0_) and local shape variations located towards the outer circle (L_5_).

Starting from 3D landmark heritability and 3D pairwise landmark co-heritability (Figure 2) in children, we subdivided facial shape into 63 modules of co-inheritance (Figure 3). First, the midface was separated from the rest of the face and was further partitioned into the philtrum area (quadrant 3, starting at segment 6) and nose (quadrant 4, starting at segment 7). The same was supported by the facial maps of co-heritability (Figures 2A,B,E) in which the nose is presented as an autonomous feature. The remainder of the face was decomposed into the lower facial area (quadrant 1, starting at segment 4) and upper facial area (quadrant 2, starting at segment 5), also promoted by the co-heritability maps in Figures 2A,H. Each segment was repeatedly partitioned into two toward the next level, increasingly focusing on local shape variations. The structural modules are depicted in Supplementary Figure S2 and a substantial overlap between the two alternative segmentations was proven by the high NMI scores (NMI_L0_ = 1, NMI_L1_ = 0.76, NMI_L2_ = 0.84, NMI_L3_ = 0.77, NMI_L4_ = 0.76, NMI_L5_ = 0.73).

### Modular Heritability

**FIGURE 4:**
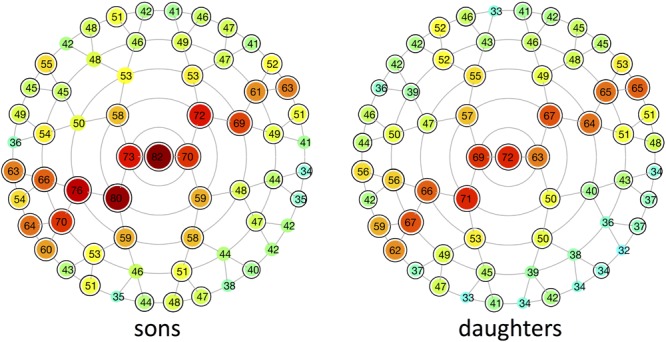
Heritability of different global-to-local parts in the face. Modular heritability estimates (%) for sons and daughters, as obtained from the regression on fathers. Each node corresponds to the facial segments depicted in Figure 3. The red-blue spectrum represents levels of high and low heritability, respectively, and the corresponding values are plotted on top of each node. Black-encircled facial segments had *p*-values below the significance threshold correcting for the multiple-testing burden (α = 1.3889 × 10^-3^). All significance tests were based on 1,000,000 permutations.

Multivariate estimates of heritability were obtained for all 63 modules in sons and daughters separately (Figure 4). Nearly all modules reached statistical significance (Figure 4 and Supplementary Figure S3), displaying low (<35%) to high (>65%) heritability estimates. The corresponding MSE values are listed in Supplementary Table S2 and stabilization of the estimates is demonstrated in Supplementary Figure S4. Modules of high heritability covered well known facial areas in both cohorts, including the nose, orbital area and upper facial parts. However, higher estimates were obtained in sons compared with daughters. The lower part of the face only showed low to moderate levels of heritability, whether or not reaching the significance threshold (quadrants 1, 3). The highest level of heritability was found for the global face (segment 1), followed by the facial area encompassing the nasion, zygomas and forehead (segment 5). Heritability of the nose was also high, with the nasal bridge being more heritable than the nasal tip and alae nasi. Similar to the landmark-based approach (Figure 1), heritability of the philtrum (segments 6, 12, 24) was lower in daughters compared with sons, whereas modular estimates of the chin (segments 33, 34) were roughly the same in both cohorts.

**FIGURE 5:**
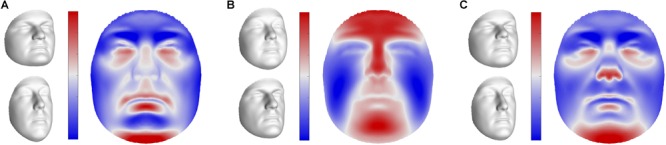
Global shape variations in offspring. Visualizations of the first three extracted latent variables at the global level in sons. Shape variations in daughters and fathers can be found in Supplementary Figure S5. **(A)** PLS component 1, **(B)** PLS component 2, **(C)** PLS component 3. In gray, illustrations of shape transformation or morph images (±4 standard deviations of the median), representing the direction in shape space encoded by the latent variables. In color, the normal displacement in each quasi-landmark, going from the lower (top) to the upper (bottom) extreme. Blue, inward repression; red, outward protrusion.

The first three pairs of latent variables were visualized at the global level in sons (Figure 5), ordered according to the percentage covariation explained. Additional figures representing the corresponding directions in daughters and fathers can be found in Supplementary Figure S5. The first pair included aspects related to facial roundness in both fathers and children (Figure 5A and Supplementary Figure S5A), e.g., a short and round face versus an elongated face. The second and third latent pairs represented variation in the prominence of the midfacial area (Figure 5B and Supplementary Figure S5B), e.g., protrusion versus retrusion, and the angle of the nasal tip and prominence of the chin (Figure 5C and Supplementary Figure S5C), respectively.

## Discussion

In this study, we present a novel approach to determine facial heritability and co-heritability in fathers and offspring, starting from 3D spatially dense descriptions of the human face. By combining the co-heritability of neighboring quasi-landmarks, novel phenotypes with particular heritabilities were generated based on the biological mechanisms of inheritance. In addition to the analysis of co-inheritance, a second novel aspect of this work is the multivariate investigation of facial shape at multiple levels of organization. In general, similar patterns of facial features were inherited from the fathers to their 15-year-old offspring, including the global face, upper facial parts (forehead, orbital region, nasion) and the nose. Interestingly, sons showed stronger heritability to their fathers than daughters did at the age of 15.

### Multivariate Analysis of Facial Shape

In contrast to previous work where facial shape is only represented using a sparse set of anatomical landmarks (Tables 1–3), spatially dense representations provide detailed information on the entire geometry of the facial surface (Claes et al., 2012a; Tsagkrasoulis et al., 2017). Moreover, the modularization approach allows focusing on more local shape variations in PCA space. Some previous studies also used PCA (Tables 1–3), but heritability scores were computed for each of these PCs separately. However, orthogonality in the PCs typically overlooks the underlying covariance structure. By contrast, the full-range of facial variations was retained throughout our analyses by considering all PCs simultaneously in a multivariate regression approach. Furthermore, preselection of facial traits as univariate measurements may introduce artifacts and results need to be interpreted with care. Analysis of heritability in the x-dimension consistently showed higher values of heritability at the midline (Supplementary Figure S6), which were erroneously introduced by aligning the faces at x = 0 during GPA, leading to an almost absence of variation in this region (i.e., order of 10^-22^). The same can be observed in the work of Tsagkrasoulis et al. (2017), where high levels of heritability seem to overlap with regions of zero-curvature, for example at the nasolabial folds.

### Novel Facial Phenotypes Determined From the Patterns of Inheritance

A limitation of the AM mapping in combination with GPA to establish spatially dense configurations, is that quasi-landmarks are positioned in the context of all other quasi-landmarks. Facial areas comprising of densely sampled points, e.g., the cheeks, will hence drive the superimposition. This will also affect the heritability estimates obtained in Figure 1, although highlighted regions in our study still coincided with genetically determined facial traits as previously reported in literature (Tables 1–3). The modularization approach surpasses this limitation because all modules are subjected to a separate GPA. In this way, only biological shape was captured, independent from its integration within the full face. Yet, information on the integration of facial parts at higher levels was preserved through the hierarchical construction.

The hierarchical clustering approach is data-driven and segmentation of the face was performed on data of sons and daughters combined to increase the number of individuals (*N* = 762), although differences in patterns of inheritance were observed between the two cohorts. As expected, the degree of overlap between the co-heritability-based and structural segmentation was high because genetic and functional mechanisms are likely to be linked. Interestingly, modules of co-inheritance seem to reflect differences in tissue types. To illustrate, segment 9 coincided with the maxillary bone (Figure 3), whereas the same segment represented the mouth in the structural approach (Supplementary Figure S2). The nasal tip and bridge (segments 57 and 58, respectively) also formed distinct modules, reflecting the underlying bone-cartilage framework. This result is consistent with the one depicted in Figure 2E and imposes well-known difficulties in forensic applications such as craniofacial reconstruction (Claes et al., 2010).

### Estimating Different Components of Variance

It is important to realize that heritability is only a descriptive statistic, referring to a particular population under particular conditions (Falconer and Mackay, 1996; AlKhudhairi and AlKofide, 2010). The reported heritability estimates were based on a European cohort and may not generalize to other populations. Various methods have been used to quantify facial heritability and as with other designs based on relatives, genetic contributions computed here are likely to be biased upward due to the environmental sources of covariance, i.e., common environment, providing an upper bound for the heritability. In essence, heritability is always a variable and never an absolute value, hence findings across studies should be interpreted in terms of low, moderate and high heritability only.

Mixed models provide an alternative method to estimate variance components, allowing for interactions between genotype and environment (Visscher et al., 2008; Lange et al., 2016; Mayhew and Meyre, 2017). Moreover, they can handle different types of (genetic) relationships as well. Moving toward the post-GWAS era, heritability studies will likely shift from the classical twin and family designs toward approaches estimating phenotypic variance from genome-wide SNP (i.e., single nucleotide polymorphism) data, as in the recent population study of Cole et al. (2017). In the context of ‘global’ shape heritability, Klingenberg (2003) states that only the GP^-1^ matrix (where G and P are the genetic and phenotypic covariance matrices, respectively) can be regarded as the multivariate equivalent of the otherwise univariate heritability measure because the spatial structure of variation is ignored when Procrustes distances are used (cf. Monteiro et al., 2002). The latter is only justified if the assumption of model isotropy holds or if the P and G matrices are proportional (Klingenberg and Monteiro, 2005). However, variance component analyses using mixed models are currently difficult to implement for high-dimensional data due to the computational burden. Moreover, patterns of shape variations in fathers correlated well with those predicted in children (Figure 5 and Supplementary Figure S5).

### Facial Heritability and Co-heritability of Different Global-to-Local Segments

Low (<35%) to high (>65%) heritability estimates were obtained for different global-to-local parts in the face, ranging from 32 to 82%. As it is difficult to compute the appropriate sample size in our multivariate approach, we ran an analysis to experimentally determine the effect of sample size on the estimates. From Supplementary Figure S4 we can conclude that, for all segments, the heritability converges toward the tail end of the curve and stable estimates are generated.

In contrast to previous parent-offspring studies on craniofacial heritability, higher estimates were obtained in sons compared with daughters for the majority of the facial segments (Table 2). Heritability of the global face was maximal and equal to 82 and 72% in sons and daughters, respectively, higher than the global estimate observed by Cole et al. (2017) (Table 3). Given the remarkable facial similarity between first-degree relatives, such high values are expected. Consistent with the literature, genetic determination was found for midfacial parameters (Tables 1–3). High heritability was observed for nasal structures in both 3D landmark- and modular-based approaches (Figures 1, 4), as confirmed by previous heritability studies (Tables 1–3). In particular, there is general agreement on the high heritability of the position of the nasion, which is strongly linked to the *PAX3* gene (Liu et al., 2012; Paternoster et al., 2012; Adhikari et al., 2016; Claes et al., 2018). Similar to previous studies who reported high heritability of intercanthal width and other traits related to the orbital region (Tables 1–3), we also observed high heritability of the corresponding segments (segments 21, 43). Strong genetic control was found for the upper facial part in general (segment 5), encompassing the zygomas and forehead in addition to the nasion and orbital structures. There have been very few studies investigating the heritability of the forehead, mainly because measurements in this region are lacking due to the definition of anatomical landmarks in the midface only. Surface shape analysis in twins revealed intrapair similarities in the region of the forehead (Naini and Moss, 2004; Djordjevic et al., 2013), consistent with the present results and the study of Tsagkrasoulis et al. (2017) (Table 1).

As expected, heritability of the lower parts of the face only ranged from low to moderate. The effect of BMI or facial fatness is mainly located in the areas around the cheek, chin and neck, reflecting the greater environmental component (Shrimpton et al., 2014). Moreover, there is a greater chance of movement of the jaw as well as a greater risk of trauma. The mandible is also influenced by function, e.g., breathing and eating habits (Al Ali et al., 2014a,b, 2015), and possible middle ear infections might interfere with the growth of the mandible (Kaneyama et al., 2008). In this study, low heritability was specifically observed in the small segments around the philtrum (segments 48–55) and cheeks (segment 32), whether or not significant. A number of genes associated with lip morphology have previously been identified (Wilson-Nagrani, 2016), yet differing levels of heritability were reported in literature (Tables 1–3). Moderate heritability of the mouth (segment 38) was found in this study. Facial segments around the chin area displayed similar levels of moderate heritability in both cohorts (cf. Lahoti et al., 2013; Tsagkrasoulis et al., 2017; Tables 1, 2), whereas 3D landmark heritability in the same region was higher in daughters compared with sons. This inconsistency between the two approaches may be related to the superimposition step as mentioned before, because integration-effects may still be present in the landmark-based approach. In addition, differences in our 15-year-old study cohort can partly be explained by gender-related differences in facial maturation. It is acknowledged that facial maturity develops in women between 12 and 14 years and 2 years later for men, hence male subjects may still be in puberty (Amini and Borzabadi-Farahani, 2009; Šidlauskas et al., 2016). Similarly, facial shape in boys continues to change between ages 12 and 16, mainly involving changes in the area of the chin, nose and supraorbital ridges (Kau and Richmond, 2008; Matthews et al., 2018). Furthermore, contributions of the mothers to the facial features of the offspring would also yield additional information enabling relative parental contributions to the facial shape in their offspring.

The regression approach of Monteiro et al. (2002) is limited in that comparison between modules of different dimensions is not straightforward, even though variation in parental and offspring phenotypes is corrected for. Therefore, multivariate correlation coefficients instead of regression coefficients were used to compute modular co-heritability (Supplementary Figure S7). Given a one-parent one-offspring design, the correlation is the same as the regression when variances in parental and offspring values are equal (Falconer and Mackay, 1996). However, this assumption is often not met. In our example, the moderate to high levels of correlation between the four quadrants (Supplementary Figure S7, level 2) further supported the high level of heritability of the global face.

### Heritability Perspective on 3D Facial Shape in Practice

In sharp contrast with the high heritability, little is known on the genetic determinants of particular facial features (Roosenboom et al., 2016). Knowledge of which part of the facial surface is under strong genetic control and which part is mostly influenced by other factors like environmental influences or gene-environment interactions is useful in genetic association studies and allows focusing on those facial parameters displaying a strong genetic component. This can be confirmed by the association study of Claes et al. (2018), where global and local facial patterns of the discovered loci involved modules that are reported here as moderate to highly heritable, e.g., the nose, chin and forehead. Information on modular co-heritability is also useful in the definition of facial phenotypes. The hierarchical clustering approach forces the decomposition of modules into two more localized segments, but preservation of the original segment might be favorable when co-heritability is high. In addition to the use in association studies, the heritability perspective on 3D facial shape is also relevant for a variety of other scientific disciplines, such as anthropology, dysmorphology, ophthalmology, otolaryngology (ENT), orthodontics, craniofacial surgery and forensics.

## Conclusion

In conclusion, we here propose a multivariate framework to explore genetic and environmental contributions to facial shape in families (grandparents, parents, and their offspring), which is of interest in a number of fields that deal with craniofacial morphology. Segmentation of the face into modules of co-inheritance allows focusing on global and local aspects of facial variation, demonstrating evidence of high heritability for the global face and for midfacial structures, such as the nasal and orbital region, in both sons and daughters.

## Data Availability Statement

The dataset analyzed for this study (B2409: “Exploring the heritability of facial features in fathers and offspring using spatially-dense geometric morphometrics”) can be requested through the ALSPAC website. Please refer to the ALSPAC access policy for further details: https://www.bristol.ac.uk/alspac/researchers/data-access/.

## Author Contributions

HH drafted the manuscript and performed all analyses under the supervision of PC, HP, and GH. PC and SR were involved in the initial project design and SR with AZ coordinated the collection of the children’s images. PC, JL, and KI provided input on the application of the analyses. DG conceptualized and implemented the connectome plot containing modular heritability and co-heritability. PC, HP, GH, SR, and ML aided in the interpretation of the data and revised the manuscript. All authors contributed to read and approved the final manuscript.

## Conflict of Interest Statement

The authors declare that the research was conducted in the absence of any commercial or financial relationships that could be construed as a potential conflict of interest.

## Footnotes

1 http://www.bris.ac.uk/alspac/researchers/data-access/data-dictionary/

2 The facial mapping software is available at https://github.com/TheWebMonks/meshmonk, free to use for academic purposes.
