# Supplementary material for: Spatially Dense 3D Facial Heritability and Modules of Co-heritability in a Father-Offspring Design
Source: Front Genet. 2018 Nov 19;9:554. doi: 10.3389/fgene.2018.00554 (PMC6252335; doi:10.3389/fgene.2018.00554)
Supplement: Supplementary file 1 [file Data_Sheet_1.PDF]

Supplementary Material

Spatially-Dense 3D Facial Heritability and Modules of Co-Heritability in a Father-Offspring Design

Hanne Hoskens*, Jiarui Li, Karlijne Indencleef, Dorothy Gors, Maarten H.D. Larmuseau, Stephen Richmond, Alexei I. Zhurov, Greet Hens, Hilde Peeters, Peter Claes*

*** Correspondence:** Hanne Hoskens: hanne.hoskens@kuleuven.be;
Peter Claes: peter.claes@kuleuven.be

1 Supplementary Figures

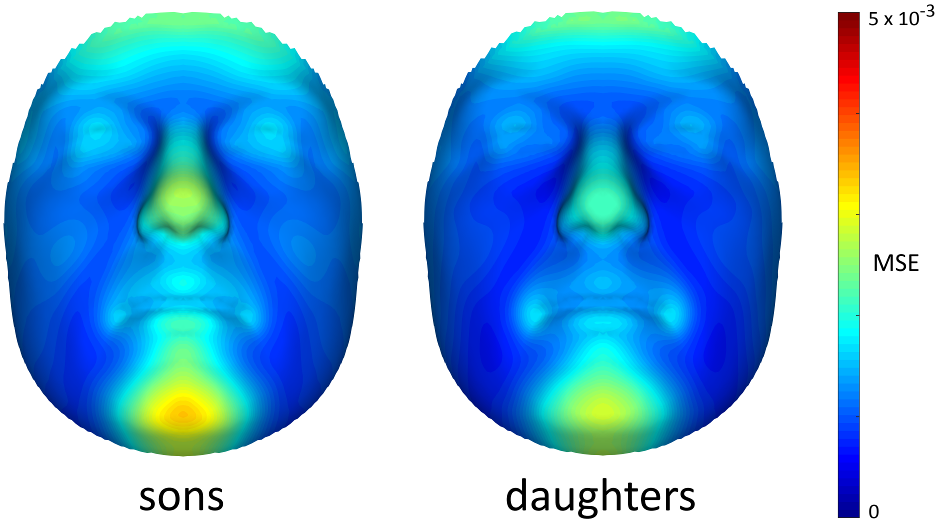

Supplementary Figure 1. Mean squared error of the 3D landmark regression models. MSE values of the PLSR model for each landmark in sons and daughters. The red-blue spectrum represents regions of larger and smaller errors, respectively.
