# Supplementary material for: Spatially Dense 3D Facial Heritability and Modules of Co-heritability in a Father-Offspring Design
Source: Front Genet. 2018 Nov 19;9:554. doi: 10.3389/fgene.2018.00554 (PMC6252335; doi:10.3389/fgene.2018.00554)
Supplement: Supplementary file 2 [file Data_Sheet_2.PDF]

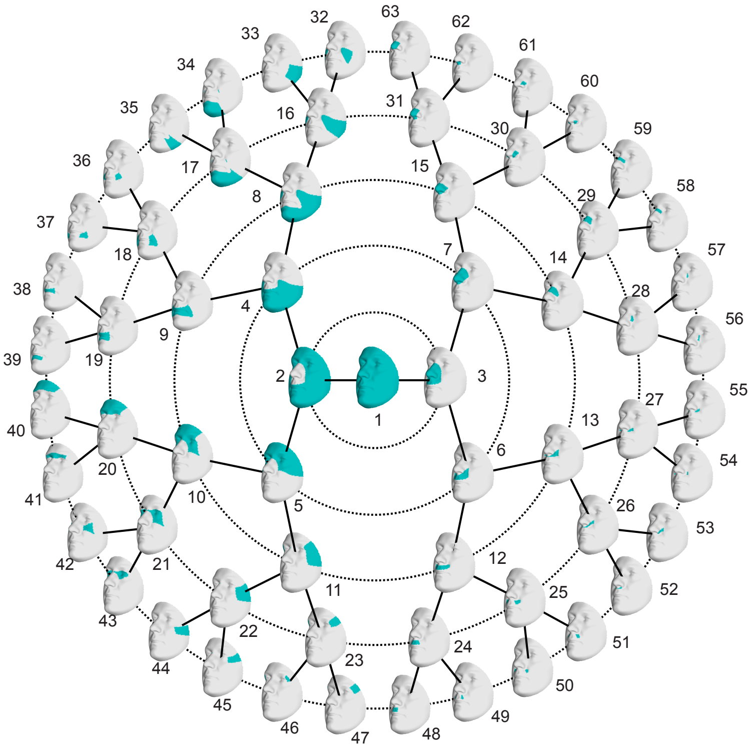

Supplementary Figure 2. Structural modules. Hierarchical facial segmentation of the study cohort, resulting from the grouping of highly correlated quasi-landmarks in children (N = 762 subjects). Segments are colored in blue. Facial shape variation is covered at five different levels of detail, with global shape variations located in the center (L_0) and local shape variations located towards the outer circle (L_5).
