# Supplementary material for: Spatially Dense 3D Facial Heritability and Modules of Co-heritability in a Father-Offspring Design
Source: Front Genet. 2018 Nov 19;9:554. doi: 10.3389/fgene.2018.00554 (PMC6252335; doi:10.3389/fgene.2018.00554)
Supplement: Supplementary file 3 [file Data_Sheet_3.PDF]

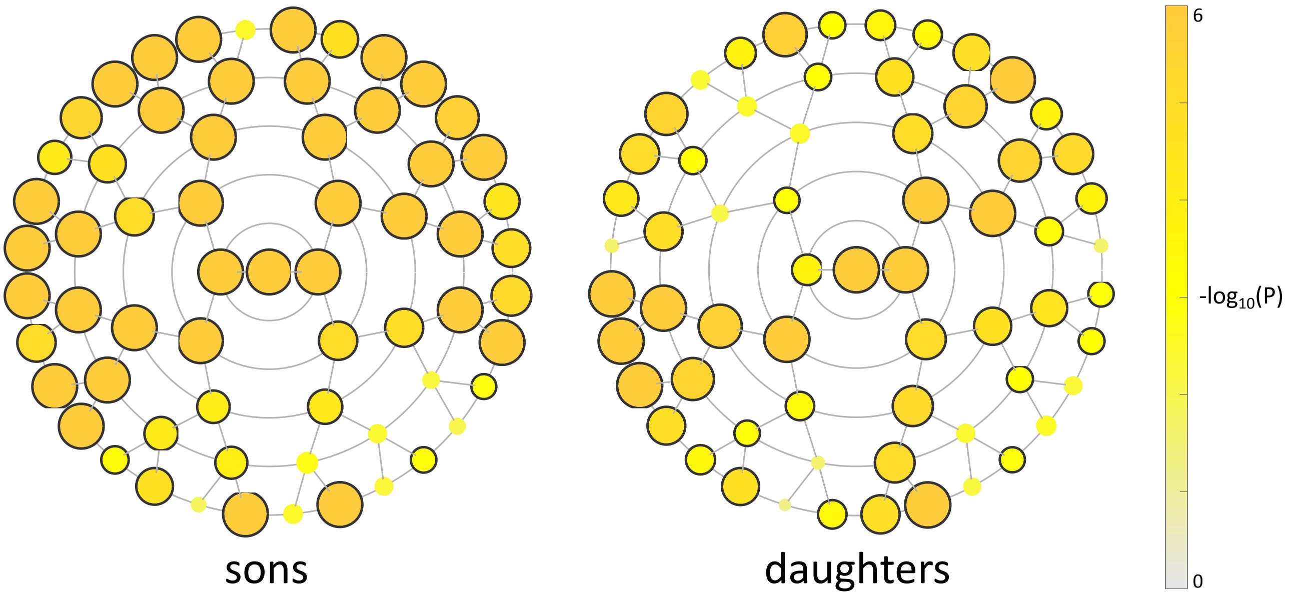

Supplementary Figure 3. Permutation testing. $-\log_{10}$(P-value) of the heritability estimates per facial segment for sons and daughters. Each node corresponds to the facial segments depicted in **Figure 3**. Black-encircled facial segments had p-values below the significance threshold correcting for the multiple-testing burden ($\alpha = 1.3889 \times 10^{-3}$). All significance tests were based on 1,000,000 permutations.
