# Supplementary material for: Spatially Dense 3D Facial Heritability and Modules of Co-heritability in a Father-Offspring Design
Source: Front Genet. 2018 Nov 19;9:554. doi: 10.3389/fgene.2018.00554 (PMC6252335; doi:10.3389/fgene.2018.00554)
Supplement: Supplementary file 4 [file Data_Sheet_4.PDF]

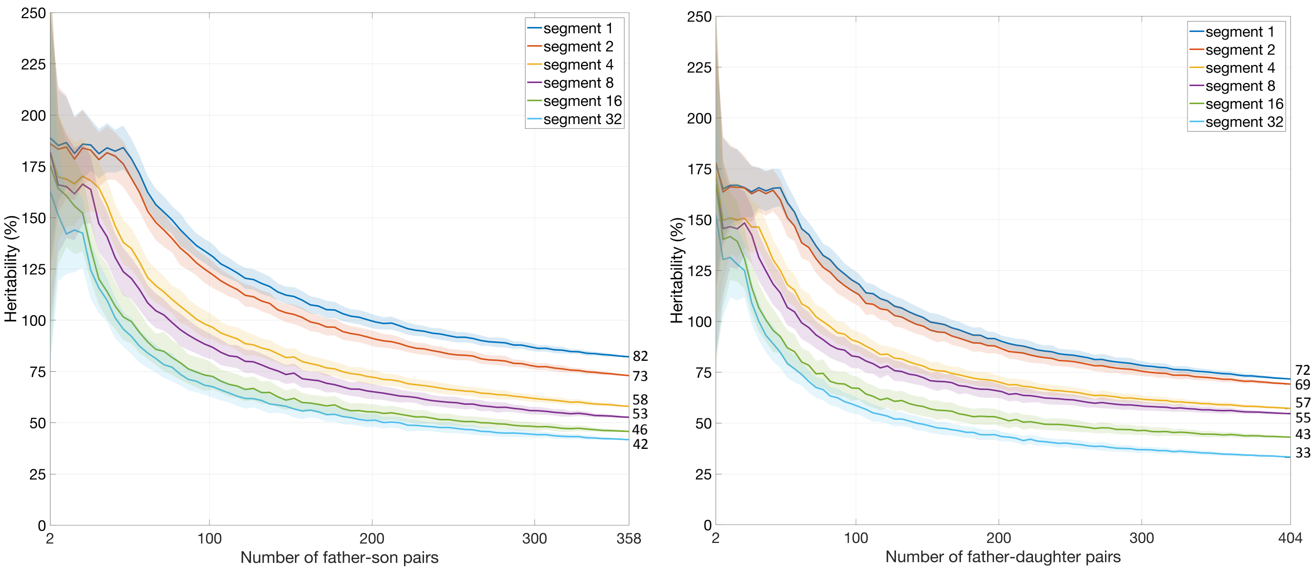

Supplementary Figure 4. Heritability estimation versus sample size. Going from the minimum (N=2) to the maximum number of father-child pairs (sons, N=358; daughters, N=404), the heritability was computed for various facial segments, representing all five hierarchical levels (**Figure 3**). This was repeated 1,000 times, each covering a different random subset. The mean heritability was plotted as a solid line and the standard deviation was plotted as the shaded area around the mean. The overall heritability estimates, computed from the maximum number of father-child pairs, are listed on the right.
