# Supplementary material for: Spatially Dense 3D Facial Heritability and Modules of Co-heritability in a Father-Offspring Design
Source: Front Genet. 2018 Nov 19;9:554. doi: 10.3389/fgene.2018.00554 (PMC6252335; doi:10.3389/fgene.2018.00554)
Supplement: Supplementary file 5 [file Data_Sheet_5.PDF]

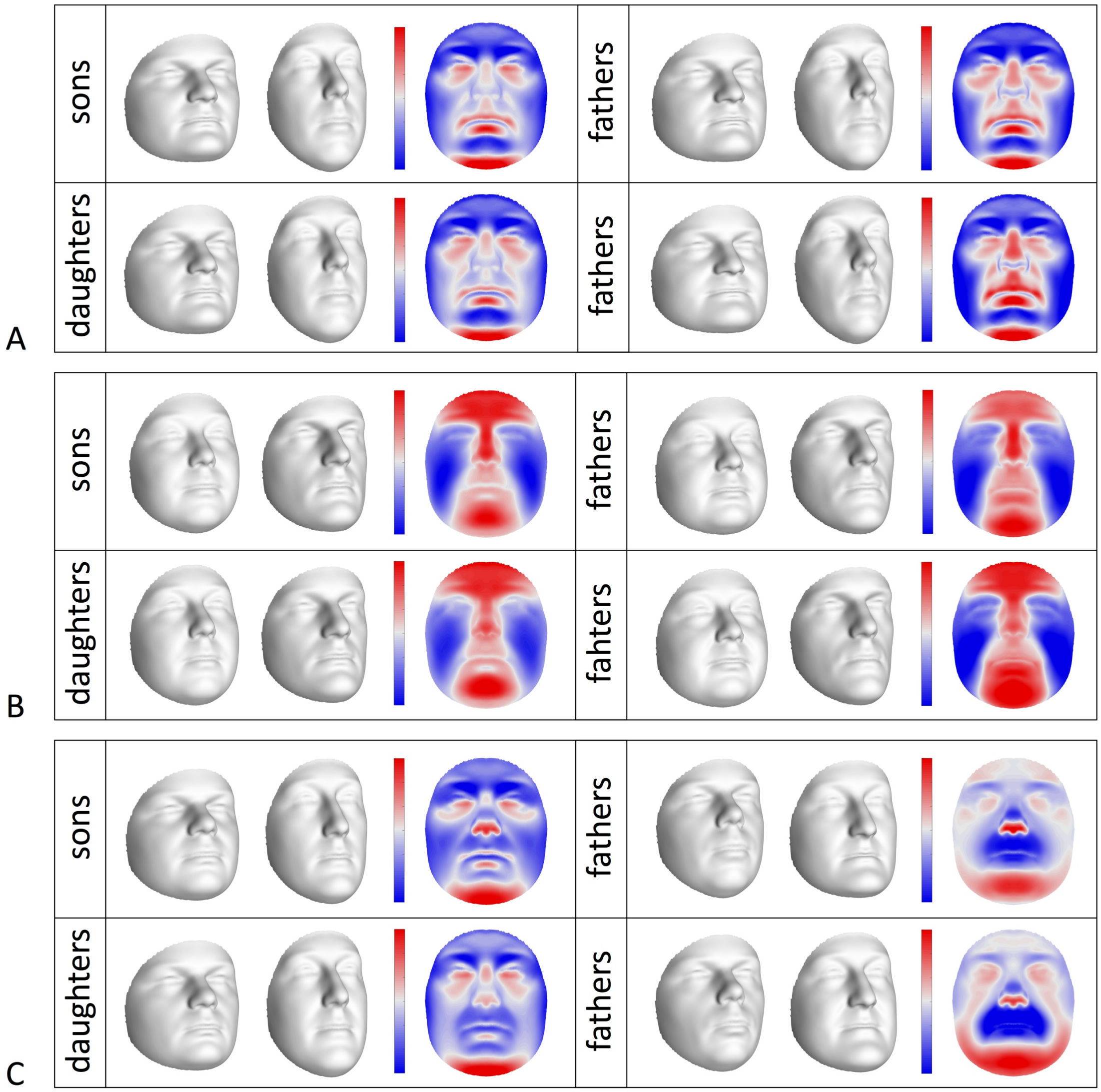

Supplementary Figure 5. Global shape variations in fathers and offspring. Visualizations of the first three extracted latent variables at the global level in fathers and offspring. (**A**) PLS component 1, (**B**) PLS component 2, (**C**) PLS component 3. In grey, illustrations of shape transformation or morph images (± 4 standard deviations of the median), representing the direction in shape space encoded by the latent variables. (right) In color, the normal displacement in each quasi-landmark, going from the lower (left) to the upper (middle) extreme. Blue, inward repression; red, outward protrusion.
