# Supplementary material for: Spatially Dense 3D Facial Heritability and Modules of Co-heritability in a Father-Offspring Design
Source: Front Genet. 2018 Nov 19;9:554. doi: 10.3389/fgene.2018.00554 (PMC6252335; doi:10.3389/fgene.2018.00554)
Supplement: Supplementary file 6 [file Data_Sheet_6.PDF]

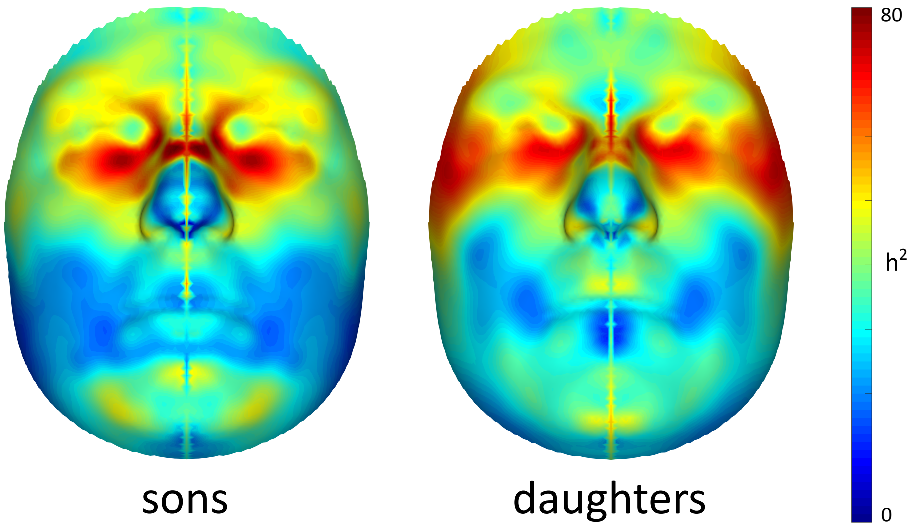

Supplementary Figure 6. Facial heritability maps in the x-dimension. Landmark (x-coordinate) heritability (%) for sons and daughters as obtained from the regression on fathers. The red-blue spectrum represents regions of high and low heritability, respectively. The maximum value was set to 80% for visualization purposes. The artifact at the midline is introduced by the alignment of the faces at x = 0 during GPA. Variation in this region is close to zero, resulting in a bias of the heritability estimates.
