# Supplementary material for: Spatially Dense 3D Facial Heritability and Modules of Co-heritability in a Father-Offspring Design
Source: Front Genet. 2018 Nov 19;9:554. doi: 10.3389/fgene.2018.00554 (PMC6252335; doi:10.3389/fgene.2018.00554)
Supplement: Supplementary file 7 [file Data_Sheet_7.PDF]

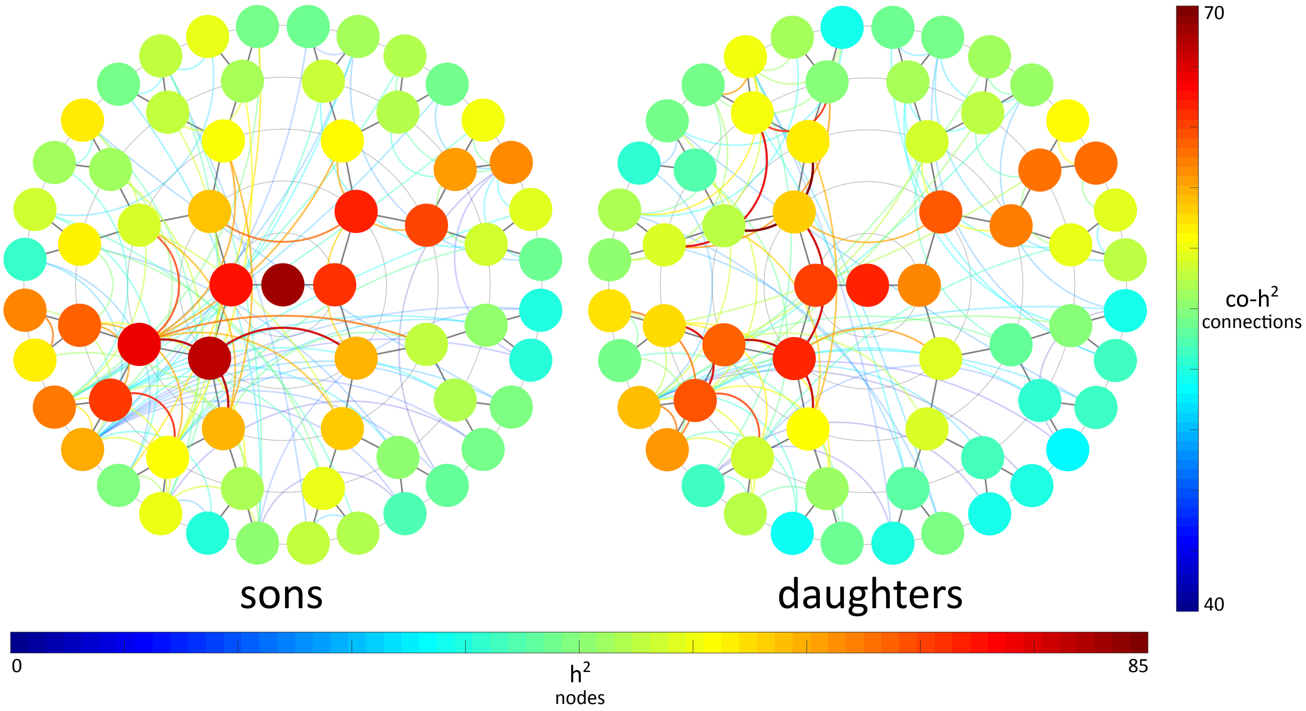

Supplementary Figure 7. Heritability and co-heritability of different global-to-local parts in the face. Modular heritability (nodes, %) in sons and daughters, as obtained from the regression on fathers. Each node corresponds to the facial segments depicted in **Figure 3**. From each facial segment, connections are drawn to the two segments that have maximal correlation with the segment being tested, within the same hierarchical level. The red-blue spectrum represents levels of high and low heritability and co-heritability, respectively.
