# Supplementary material for: Spatially Dense 3D Facial Heritability and Modules of Co-heritability in a Father-Offspring Design
Source: Front Genet. 2018 Nov 19;9:554. doi: 10.3389/fgene.2018.00554 (PMC6252335; doi:10.3389/fgene.2018.00554)
Supplement: Supplementary file 8 [file Data_Sheet_8.PDF]

Supplementary Material

Spatially-Dense 3D Facial Heritability and Modules of Co-Heritability in a Father-Offspring Design

Hanne Hoskens*, Jiarui Li, Karlijne Indencleef, Dorothy Gors, Maarten H.D. Larmuseau, Stephen Richmond, Alexei I. Zhurov, Greet Hens, Hilde Peeters, Peter Claes*

*** Correspondence:** Hanne Hoskens: hanne.hoskens@kuleuven.be;
Peter Claes: peter.claes@kuleuven.be

1 Supplementary Tables

Supplementary Table 1. Summary statistics for age and BMI in fathers and offspring.

	**age (years)**			**BMI**		
	fathers	sons	daughters	fathers	sons	daughters
mean	53.1	15.4	15.4	27.4	20.5	21.3
SD	5.1	0.2	0.2	3.8	2.9	3.5
1st quartile	50.0	15.3	15.3	24.7	18.7	18.9
median	52.0	15.3	15.3	26.9	20.0	20.6
3rd quartile	56.0	15.4	15.5	29.4	21.7	23.0
min	40.0	14.5	14.8	18.9	14.9	15.1
max	75.0	16.3	16.9	43.7	34.8	36.8

fathers (N = 762), sons (N = 358), daughters (N = 404)
SD, standard deviation; min, minimum value; max, maximum value
