# Supplementary material for: Spatially Dense 3D Facial Heritability and Modules of Co-heritability in a Father-Offspring Design
Source: Front Genet. 2018 Nov 19;9:554. doi: 10.3389/fgene.2018.00554 (PMC6252335; doi:10.3389/fgene.2018.00554)
Supplement: Supplementary file 9 [file Data_Sheet_9.pdf]

Supplementary Table 2. Mean squared error of the modular regression models.

(*) non-significant heritability estimates. MSE, mean squared error.

Facial segmentation		Sons		Daughters	
level	segment	h^2 (%)	MSE	h^2 (%)	MSE
0	1	82	8.40	72	7.70
1	2	73	6.73	69	6.11
	3	70	2.72	63	2.51
2	4	58	4.15	57	3.55
	5	80	2.68	71	2.37
	6	59	0.84	50	0.76
	7	72	1.84	67	1.67
3	8	53*	2.78	55	2.36
	9	50*	1.27	47	1.14
	10	76	2.99	66	2.40
	11	59	0.54	53	0.49
	12	58	0.63	50	0.60
	13	48	0.16	40	0.14
	14	69	1.13	64	1.04
	15	53	0.41	49	0.34
4	16	46	0.57	43	0.48
	17	48*	2.07	52	1.76
	18	45	0.11	39	0.09
	19	54	0.92	50	0.84
	20	66	1.63	56	1.32
	21	70	0.92	67	0.77
	22	53	0.20	49	0.19
	23	46*	0.14	45	0.11
	24	51	0.48	39*	0.46
	25	44*	0.13	38*	0.13
	26	47	0.04	36*	0.04
	27	44	0.05	43	0.04
	28	49	0.90	51	0.85
	29	61	0.81	65	0.72
	30	47	0.05	48	0.04
	31	49	0.28	46	0.23
5	32	42	0.17	33*	0.14
	33	51	0.49	46	0.40
	34	48	1.39	52	1.22
	35	42*	0.23	42	0.19
	36	55	0.04	42	0.03
	37	45	0.03	36	0.03
	38	49	0.84	46	0.73
	39	36*	0.08	44	0.08
	40	63	1.41	56	1.08
	41	54	0.16	42	0.13
	42	64	0.16	59	0.13
	43	60	0.56	62	0.52
	44	43	0.06	37	0.05
	45	51	0.07	47	0.06
	46	35*	0.03	33*	0.03
	47	44	0.04	41	0.04
	48	48	0.19	34*	0.18
	49	47	0.18	42	0.16
	50	38*	0.08	34*	0.08
	51	40	0.04	34	0.04
	52	42*	0.02	32*	0.02
	53	42*	0.01	37	0.01
	54	35	0.01	37	0.01
	55	34	0.02	34	0.01
	56	41*	0.29	48	0.26
	57	51	0.51	51	0.50
	58	63	0.58	65	0.54
	59	52	0.10	53	0.09
	60	41	0.02	45	0.02
	61	47	0.01	45	0.01
	62	46	0.20	42	0.17
	63	41	0.05	41	0.04
